# When feathers fell from fashion: unpacking the end of the UK plumage trade

**DOI:** 10.1098/rsos.252133

**Published:** 2026-10-21

**Authors:** Sally L. Sinclair, Audrey Dickinson, Diogo Veríssimo

**Affiliations:** ^1^ Durrell Institute of Conservation and Ecology, University of Kent, Canterbury, UK; ^2^ Department of Science and Conservation, San Diego Botanic Garden, Encinitas, CA, USA; ^3^ Salk Institute for Biological Studies, La Jolla, CA, USA; ^4^ Environment Change Institute, School of Geography and the Environment, University of Oxford, Oxford, UK; ^5^ Wildlife Conservation Research Unit, Department of Biology, University of Oxford, Oxford, UK

**Keywords:** impact evaluation, wildlife conservation, conservation policy, qualitative evaluation, behaviour change, qualitative impact assessment, causal inference

## Abstract

By the mid-twentieth century, the previously booming bird feather trade was a fraction of what it was in its prime, allowing for the recovery of many bird species. We use a qualitative impact evaluation method to assign probable causality to different drivers of this change, ultimately providing expert-supported explanations of how a large-scale behaviour change was enacted to benefit conservation. We outline a method for applying general elimination methodology plus (GEM+), accounting for different facets of evidence reliability, to generate conclusions on most strongly supported causes of change. GEM+ addresses calls for an increase in impact evaluation in conservation, which will require a range of tools, including qualitative, to fill. Analysis of diverse evidence using GEM+ suggests that conservation advocacy, interacting with broader contextual factors, plausibly contributed to a shift in public behaviour that was instrumental in the passage of the Plumage Act and the subsequent end of the trade. This case study has widespread implications for designing effective behaviour change campaigns, as well as providing a framework for conducting impact evaluations in a range of conservation settings.

## 1. Introduction

### 1.1. Impact evaluation in conservation

Over the past several decades, challenges in impact evaluation in conservation have become increasingly clear, and calls for better integration of impact evaluation have grown [1]. Given scarce resources often afforded to conservation, maximizing impact and therefore positive outcomes for biodiversity is imperative [2]. Causal inference methods can provide such answers to whether actions are generating intended impacts [3].

A key shortcoming in impact evaluations for conservation is the underutilization of qualitative methods [4], and the low appreciation of a range of evidence types to answer conservation questions [5]. While some solutions have been proposed to address this gap [4], there still exists scant application of effective evaluation in a conservation context [6]. Methods such as randomized control trials are still often considered the ‘gold standard’ in evaluation; however, they are often inapplicable to conservation contexts [7]. Given complex social factors that need to be accounted for in conservation project evaluation, the mainstreaming of evaluation in conservation will require the application of a range of methods. Outlining the repeatable use of a qualitative method here aims to contribute to plugging the conservation-evaluation gap.

We employ and improve upon existing examples of general elimination methodology (GEM [8]) to outline causal pathways through which a societal change of substantial conservation impact occurred. Using this method, rival explanations of an impact are systematically ruled out using a range of evidence including expert interviews to support or refute claims, resulting in the definition of potential causal pathways.

### 1.2. Feather fashions

In the nineteenth century, bird feathers were a widely popular fashion item. Particularly in millinery fashion, it was common for women to adorn hats with feathers of birds such as snowy egrets, or entire carcasses of smaller birds such as hummingbirds. The plumage industry was a substantial trade, with 14 362 000 lbs of exotic feathers worth £19 923 000 reportedly imported into the UK between 1901 and 1910 alone [9]. This consequently presented a global conservation issue; local extinctions of hummingbird species were noted in areas where plume hunters operated [10], and demand for great egret plumes caused an estimated 95% decline in North America (All About Birds, n.d. [11,12]). As a response in the UK, one of the largest conservation organizations globally today, the Royal Society for the Protection of Birds (RSPB), was founded with the primary goal of putting an end to the use of exotic bird plumes (i.e. plumes not of domestic origin or from ostrich) in women's fashion [13,14].

Ultimately, while there has been variation in the trade for this seasonal item, imports of feathers into the UK experienced steep decline in the late 1910s and have since remained low. Here, we evaluate potential causes of feather use decline to determine how this societal shift in behaviour occurred, enabling the recovery of multiple species.

Understanding how changes in use of wildlife products occur is key to designing future behaviour change projects and preventing damaging behaviours from reoccurring. Unsustainable wildlife trade can drive the decline of wildlife populations, and is often driven by demand that cannot be understood without considering complex social and ecological systems [15]. We apply a qualitative impact evaluation method to answer if, and how, actors representing the science stakeholder (conservationists) and society, enacted a behaviour change big enough to impact global avian conservation and policy cementing this change.

## 2. General elimination method plus: methodology

While GEM has been used to evaluate conservation initiatives previously, its application has been limited, as the choice in tools used for applying it are left up to the evaluator, with these methodological decisions affecting conclusions drawn [16,17]. Such limitations probably contribute to its lack of inclusion in discussion around available qualitative attribution methods [4]. We capitalize on the potential of this method in attributing probably causes of change, and suggest improvements that allow it to be exploited more fully by practitioners, through an elaborated, more applicable framework: GEM+.

We scrutinize different facets of evidence by considering qualities of the inputting agent (expert interviewed) to determine source and information reliability to make this method more transparent. We account for biases of the source, such as potential conflicts of interest (source reliability) and level of expertise on a topic (information reliability) when considering evidence influence over resultant confidence in a driver's role. We also allow improved actor inclusion: both stakeholder groups, or those with observable involvement in the topic; and subject experts, those with expert knowledge relevant to topics or drivers around the subject, are involved in evidence collection.

We elaborate on this method using an intuitively applicable stepwise process illustrated in figure 1, facilitating easier application of GEM+ to project evaluation. These improvements aim to improve robustness of application, while maintaining relative ease of use for a broad audience of evaluators.

**Figure 1.**
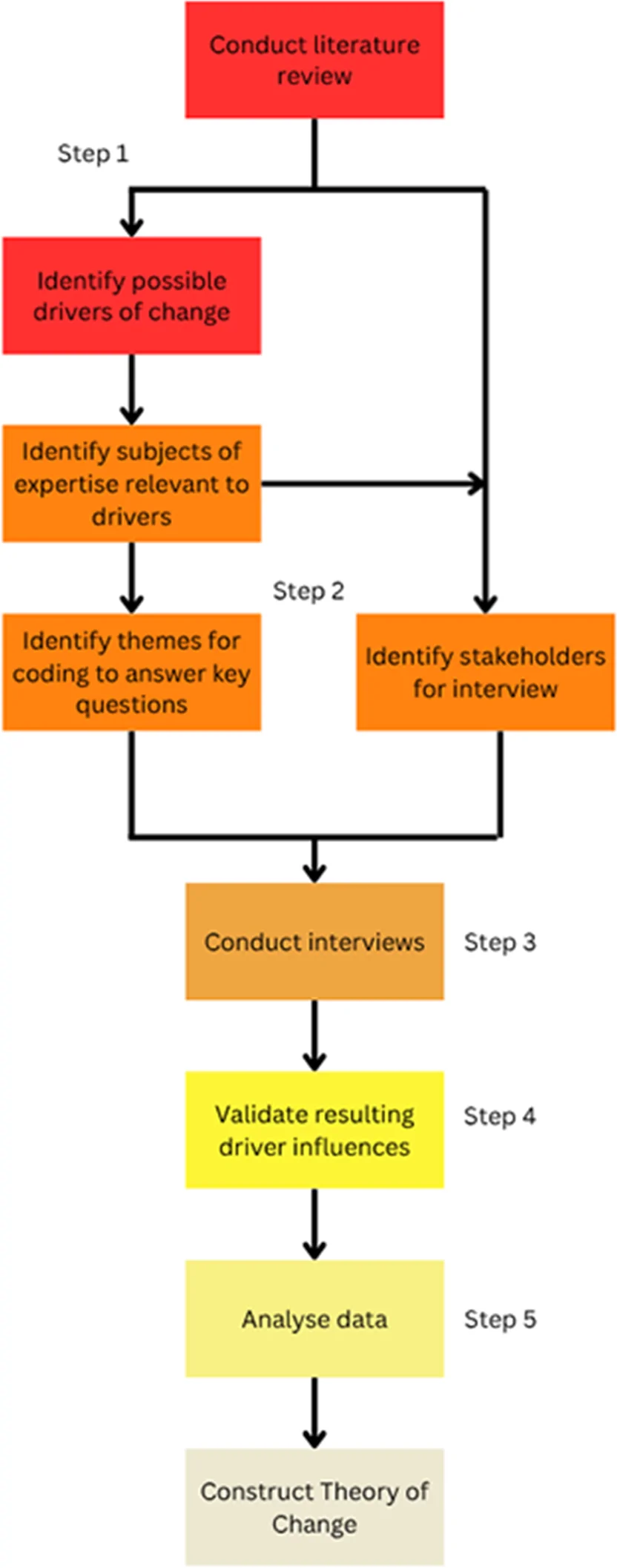
Suggested method flow for conducting GEM+.

### 2.1. Identifying drivers of change

Previously, what defines a driver has not been explicitly defined. Each potential explanation of an impact needs to be well understood to know exactly through which mechanisms, or pathways, they affected the change. We define drivers of change as distinct events or overarching contextual factors causing a direct or indirect effect on the impact of interest.

For this study, a range of sources including peer-reviewed articles, blogs, books and grey literature were investigated for factors referenced on the topic of plume decline. Supplementary material S1 outlines search terms used for finding sources and resulting reference list of sources used to identify drivers. Beginning with sources relevant to the specific topic, we identified further sources relevant to this study through backwards reference searching. The resultant list of potential drivers identified through literature review are listed in table 1.

**Table 1.** Potential drivers of changes in feather use in fashion identified through literature review.

unpredictable changes in fashion	suffragettes' movement: more political women	1921 Plumage Act
invention of the motor car	fashion changes as a result of WW1 (more utilitarian)	increased concern over the conservation of species
international anti-plumage export legislation reducing supply	economic shifts (lower consumerism and spending after WW1)	RSPB campaigning
international anti-plumage wearing campaigns reducing demand	increased access to the countryside (e.g. more bicycles)	increased animal welfare/rights sentiment

### 2.2. Identifying stakeholders and subject experts

This method considers two kinds of knowledge sources: those with a vested interest in the topic (stakeholders), and those with reliable, accurate knowledge relevant to an issue (subject experts), who may not have a vested interest in the topic.

Initially, stakeholder groups are identified by considering the reach of the topic. Given the historic nature of the change in feather use, modern representatives were required for stakeholder groups. This presents a barrier to impact evaluation for dated impacts; however, considering relevant representatives somewhat addresses this challenge. At-time stakeholders identified, considering supply and demand chains, included industry (milliners, plumassiers, fashion houses), politicians (those involved in discussions of plumage import legislation, represented by historians), those advocating against the plume trade (represented by those involved in conservation activities today) and consumers (represented by fashion historians). While some representation was lacking as a result of the historical nature of the study, that is, those with lived experience on the passing of the Plumage Act available to discuss the topic, including diverse participants to ensure a range of topic expertise addressed any potential knowledge gaps. An outline of interviewee characteristics is available in supplementary material, S2.

We then consider subject experts, who can be described as individuals that can bring unique, reliable evidence to a problem. Considering the system and impact of interest, drivers themselves provide more insight into subject expertise that could be sought on the topic, going some way to address the issue of ‘invisible’ or minority stakeholders [18]. Expertise on each driver must be present as it has been demonstrated that experts providing judgement outside their topic often do not perform substantially better than non-experts [19]. Subject experts, including academics, were included where stakeholder consultation revealed gaps in expertise on specific drivers.

Understanding each individual's expertise helps assess the accuracy of information they provide in each subject of discussion, as well as where further experts may be required to fill information gaps. Similar considerations also show where bias is high in expert responses, and where additional unbiased views need to be sought. Methods employed in expert elicitation exercises to gauge expertise level have included asking experts to rank their self-perceived level of expertise [20], allowing the interpretation of reliability of information provided by an expert on a particular topic, as well as providing insight to where there may be gaps in the expert pool. Where a high perception of expertise for any topic was not present in an existing pool of experts, this revealed a gap in expert knowledge, meaning expertise in that field needed to be included and expert pool expanded.

Source reliability was approached by assuming stakeholder groups were reliable unless clear biases were identified, resulting in an eliminative approach to groups of high source reliability. Through this, the conservation stakeholder group was removed as a source of high source reliability for rankings on the Plumage Act, anti-plumage wearing campaigns abroad, increase in concern over the conservation of species, RSPB campaigning, increased animal rights/welfare sentiment and extinction of species. This is because these topics are often directly relevant to conservation organizations' goals, meaning it would be expected that they should present these drivers as effective as a result of motivated reasoning [21]. Further to this, industry and fashion historians were removed as highly reliable sources on fashion trends as a driver, as their industries may lean towards availability bias when stating key drivers [22].

### 2.3. Interviews for evidence collection

Interviews were conducted between June and August 2024, with 15 interviews being conducted online and one in-person. For a full description of interview format, see supplementary material, S3.

Semi-structured interview design is used in GEM+. A range of experts will often be involved in applying GEM+ owing to the emphasis on representing diverse expertise, meaning accessibility of information should be considered. To open the discussion on trends in feather use in fashion, participants were shown a figure illustrating the changes in millinery fashion over the time period of interest and asked to describe the change based on this and their knowledge of millinery fashion preference for this period. This figure was designed to be accessible to a range of audiences through minimal text. Minimizing use of dates was also intended to reduce bias towards key historic events such as World War I. The period 1910–1930 was presented to focus on a strong observable change in this period, from the ‘Plume Boom’ [23] of the 1910s to post-WWI; though participants were encouraged to consider drivers before this period that may have affected the observed change.

Allowing individuals to initially self-suggest drivers reduces confirmation bias, whereby an interviewee may assume that those drivers presented to them are the most important and ignore (or forget) about others. Therefore, interviewees should be first asked to free-list potential drivers of an impact. Interviewees are subsequently presented with a list of identified drivers, excluding ones the interviewee has suggested themselves. This two-step process also reduces the amount of information interviewees have to process in each stage, reducing cognitive burden [24]. Interviewees are then asked to individually place each driver in the two lists along a scale of proposed importance in the change, the self-suggested drivers and the list presented by the interviewer were scaled separately.

Having a structured interview guide and coding sheet on how drivers incite change allows for collection of information that is more comparable across interviews. This will guide the interviewer in exactly what information they need to collect to generate a theory of change (ToC). For example, questions may focus on who the driver affected and what the immediate outcomes of driver presence were. While additional themes in coding may emerge in data analysis, this step presents a baseline for which a ToC can be developed in which causal pathway construction is more transparent and less reflective of interviewer interpretation of discussion. Our interview guide is available in supplementary material, S3. We asked interviewees to map out up to five ToCs, those ranked at a 6 or above on a scale of importance to the change. The decision to consider drivers ranked at 6 or above was chosen as a logical threshold to encompass those drivers considered most important by interviewees; all interviewees ranked at least one driver at a 6 or above. However, changing this threshold may change ultimate ToC construction—authors using this method may want to consider threshold used, for example, if there are likely to be more drivers with smaller impact to be included in a final ToC, a lower threshold may be more appropriate. Interviewees were asked to describe who was immediately affected by the driver being discussed, intermediate outcomes and facilitating contextual factors. Our coding framework including these factors, and coded information for those with self-defined expertise on the driver, is included in the online repository.

Once driver rankings and causal pathways have been discussed, information on interviewee confidence should be collected as a final interview step as outlined above in order to ascertain reliability of information provided by each individual. Information reliability for each interviewee is outlined in supplementary material, S2.

### 2.4. Validating expert input

To validate interviewee claims, external evidence is required to challenge and strengthen our understanding of the role of different drivers. Types of evidence that can be used to fill this purpose will vary [25], but should be of a primary, verifiable nature, such as peer-reviewed literature, legislative records and industry statistics. Each driver should be considered individually when collating evidence to determine what information would evidence their role in the change.

In this trade-relevant example, import statistics and quantitative information on feather popularity were sought to validate trends in feather use popularity. Information on feather imports into the UK in the period of interest was taken from Parliamentary Papers (Trade and Navigation accounts 1900–1930) and is shown in figure 2. Given the historic nature of the study, time-relevant sources were also used, including magazine experts and parliamentary discussions to build an evidence base reflecting changing attitudes and trends at the time. To gauge feather popularity in fashion, the fashion magazine *The Queen* was used given the availability of regular issues over the entire period of interest. Each fourth issue of the magazine was analysed for feather coverage, including images and text, to determine proportion of feather content in each searched issue over time. Results of this quantification of feather popularity are shown in figure 2.

**Figure 2.**
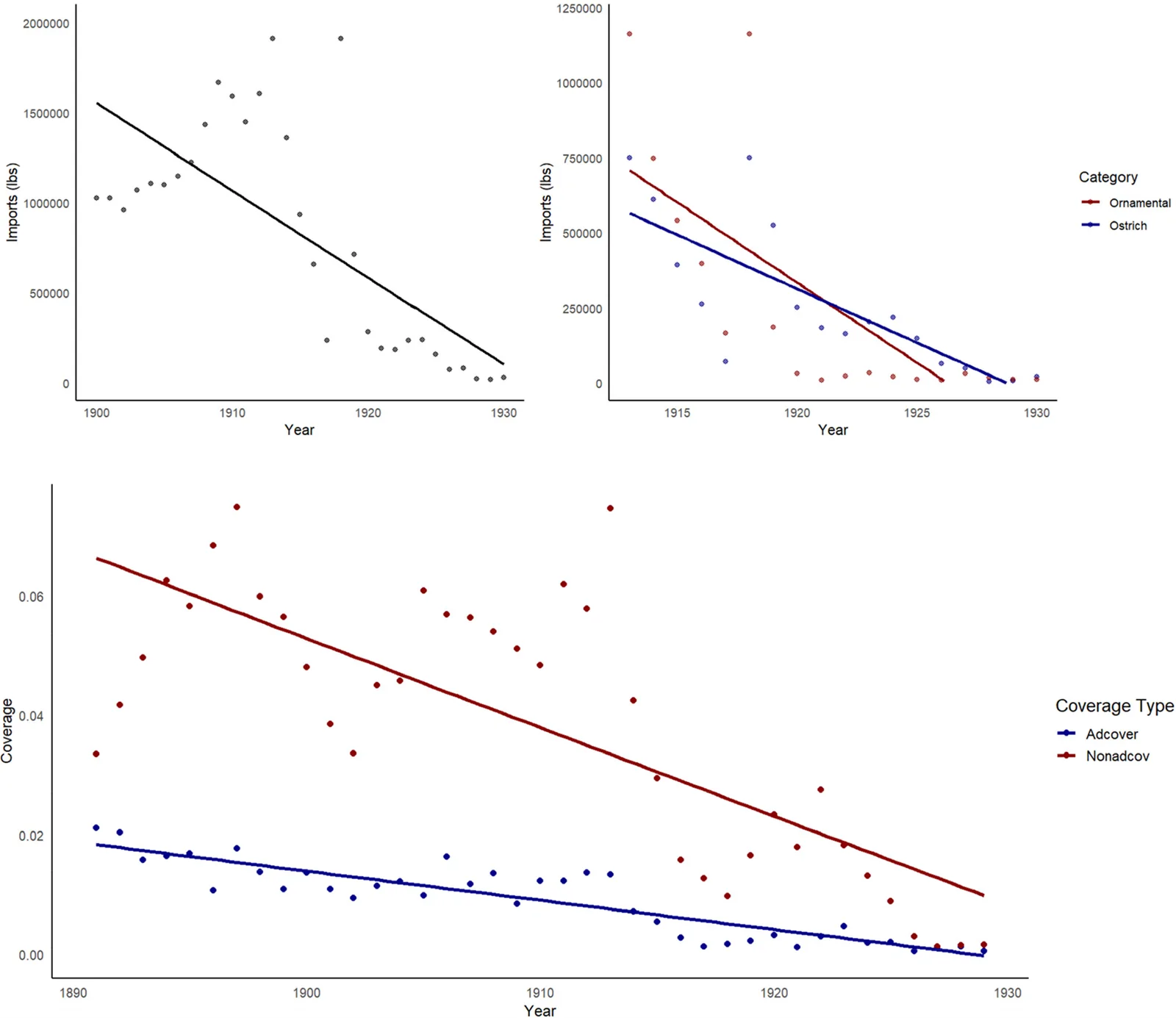
Trends in feather use over the period of interest, including [26]. (A) Importation (in lbs) of feathers, divided into ostrich and ornamental (which includes exotic plumage) into the UK, 1900–1930 and (B) proportion of *The Queen* magazine issue coverage with (1) advertisement and (2) non-advertisement feather imagery and text (1891–1930). Points show mean ad/non-ad cover for that year with line of best fit to show trends.

Additional pieces of external supporting evidence are shown in supplementary material, S4.1 and S4.2. Two individuals individually categorized these evidence bodies from ‘strongly unsupportive (1)’ to ‘strongly supportive (5)’ of that driver having played a key role in the observed change (available in supplementary material, S5). Two iterations of evidence interrogation were required to achieve inter-rater agreement of evidence support categorization.

### 2.5. Data analysis

Data analysis considers (i) driver rankings along the scale, (ii) whether group agreement on driver importance is reached, (iii) whether those individuals ranking a driver as important have high information and source reliability, and (iv) whether external evidence supports these scores.

The score a driver is assigned (through scale placement) by the expert needs to be considered to determine which drivers fall into the category of perceived highest importance by the expert. Drivers ranked at 6 or more were considered as ‘important’ for the change here.

The threshold for how many individuals in agreement define group majority also needs to be set. This could consider a proportion of the group size or a minimum number of expected inclusions by one group. The threshold for defining a group majority can also be adapted depending on inclusive power of drivers derived to a single individual depending on threshold boundaries [27]. We considered three or more individuals within a stakeholder group agreeing on driver importance to portray general support from a stakeholder group. Three was selected as the threshold here as each stakeholder group was no larger than five, meaning three individuals resulted in over 50% of the group agreeing on driver importance. For larger groups, higher thresholds may be appropriate. Equally, where greater uncertainty may be present, for example, in controversial cases, lower thresholds could also be insightful to most common responses.

Information and source reliability (defined above) improve our confidence in the evidence supporting the role of a driver in the change. Therefore, the presence of these aspects of evidence allows us to move drivers into a higher confidence group.

Interviewee judgement should be triangulated with reliable evidence sources. After removing those drivers eliminated through the interview process, supplementary material can be summarized for each driver and subsequently categorized as unsupportive, supportive or neutral of the role of the driver in the change. Evidence bodies should be ranked by multiple individuals and high agreement on categorization reached to determine the level of support provided by a body of information external to interview input. Where researchers disagree on categorization, for example, owing to mixed interpretations of trends, this should be discussed and additional information sought where necessary to reach agreement on evidence weighting.

Through each of these pieces of information, confidence in whether a driver did in fact play a role in the observed change can be determined. Figure 3 demonstrates how each of these pieces of information can be incorporated to provide an indication of confidence of driver role. Supplementary material S6 further explains the justification behind each of these confidence levels.

**Figure 3.**
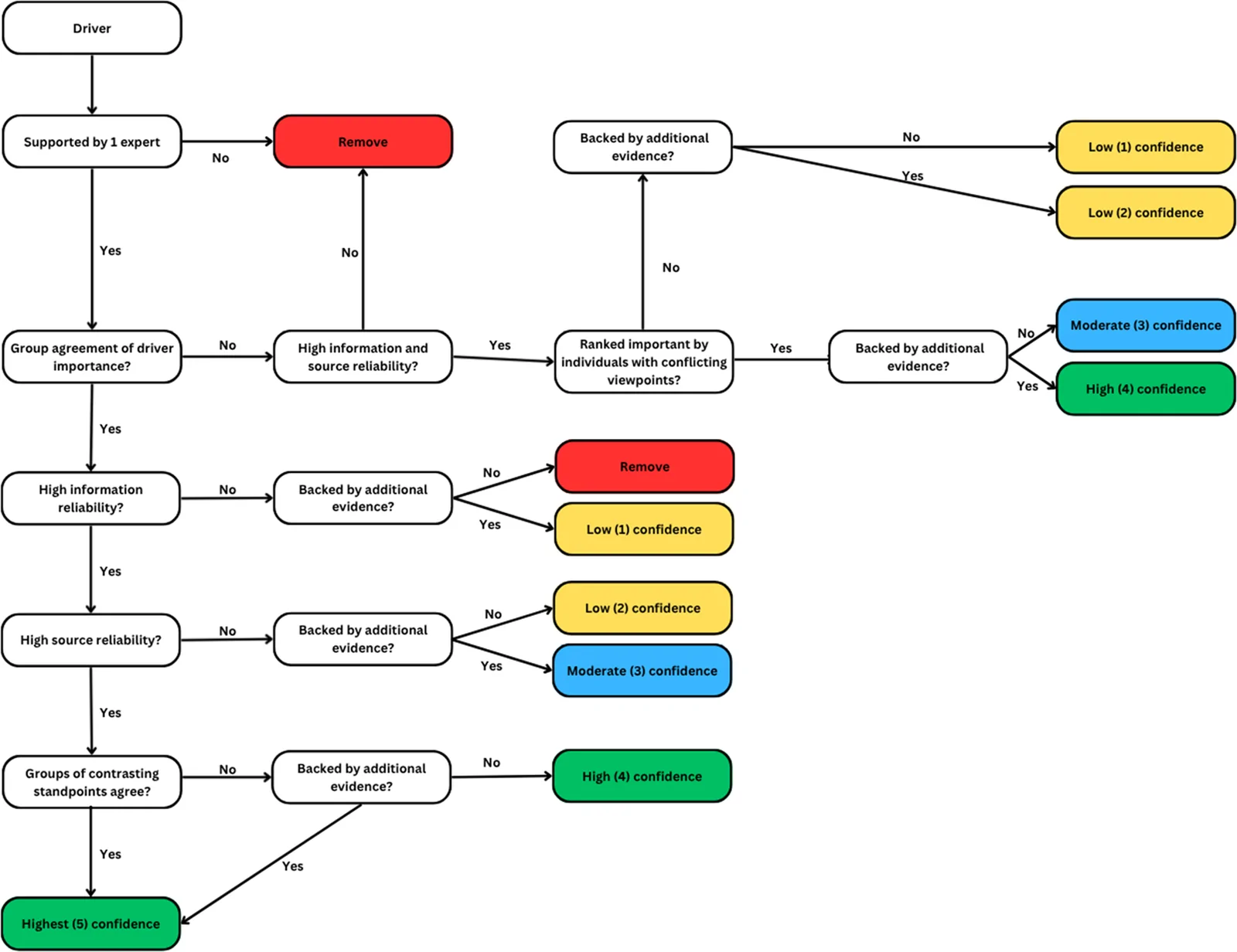
Decision pathway for driver confidence through expert elicitation and additional evidence, based on information gathered through expert elicitation and additional evidence support.

Once drivers have been categorized, they can be used to construct a ToC. As coding themes were outlined before the interviews, this should allow clearer comparison of discussion with experts to identify common ideas of how the change occurred; for example, by easily identifying how many experts listed the same stakeholder group as being directly affected by a driver and listing similar intermediate outcomes. Through this, causal pathways can be outlined. For this, information provided by those with high levels of information reliability, supported by additional resources, should be considered, owing to higher chance of inaccuracy by those with lower familiarity of the topic [19].

## 3. Results

Using the decision tree in figure 3, strength of supporting evidence for each driver derived from expert elicitation and subsequent triangulation was determined. From this, each driver was categorized into having strong, moderate or weak evidence of impact. Two drivers initially listed, namely, the invention of motor cars and increased access to the countryside, were removed as they did not meet thresholds for probably affecting the change.

Coding indicated that the reduction in plumes in fashion came about through three key pathways: (i) supply reduction and policy impact, (ii) contextual causes of change in demand, and (iii) social change in demand. These causal pathways are unpicked and described below. Quoted interviewee groups and descriptions are available in supplementary material, S2.

### 3.1. Supply reduction and policy impact

Reduction in availability to consumers was a frequent theme in the decline of the feather trade. This was primarily achieved through policy, and the Importation of Plumage (Prohibition) Act passed in 1921. The perception of relative importance of this Act is mixed, with one expert stating the Act was a case of ‘closing the stable door after the horse has bolted’ (expert C4), though many experts (seven) did rank the Act as an important factor in the change.

While a decline in imports was observed leading up to the passing of the Act, it cannot be definitively stated whether declines resulted in a reactive Act, or whether declines were in anticipation of impending legislation. Availability of individuals with lived experience on the Act may have provided more robust insight into the mechanisms through which the Act affected the feather trade. Insights from House of Lords discussions and import statistics provided some insight: imports into the UK increased slightly post-1921, and the actual enforcement of the Act and levels of smuggling are debated [28]. The Act was a result of several attempts to push plumage policy through parliament; a timely process leading to an Act described as ultimately disappointing by key advocacies for policy [29]. Key facilitating factors highlighted by experts in this pathway were the role of women, particularly in women having ‘louder voices’ for policy change, but also the male facet of the RSPB, and how these allies worked with the science and policy spheres to justify the need for policy change (see [30]).

Many experts cite international legislation as important in the decline (six), facilitated by increasing globalization and the rise of the media (figure 4). Evidence for this is mixed, with importation declines observed directly after key legislations passed by the United States, namely, the Underwood Tariff Act (1913) and the Migratory Birds Treaty Act (1918), but with impacts of legislation introduced by other nations being less apparent. The growing number of policies to impede the plume trade, supported by anti-plumage advocacy groups worldwide, would have had an impact on policy decisions in the UK. Indeed, the global nature of the trade was a frequent subject of parliamentary discussion, with concerns voiced that restricting the trade would simply move it, along with its economic benefits, to another part of Europe, with one discussion stating, ‘if this plumage trade does not find its way into this country, it will be diverted to Antwerp, Berlin and Paris, and will result not in any preservation of these birds, admirable and beautiful as they are, but merely in the transference to our foreign competitors’ [31].

**Figure 4.**
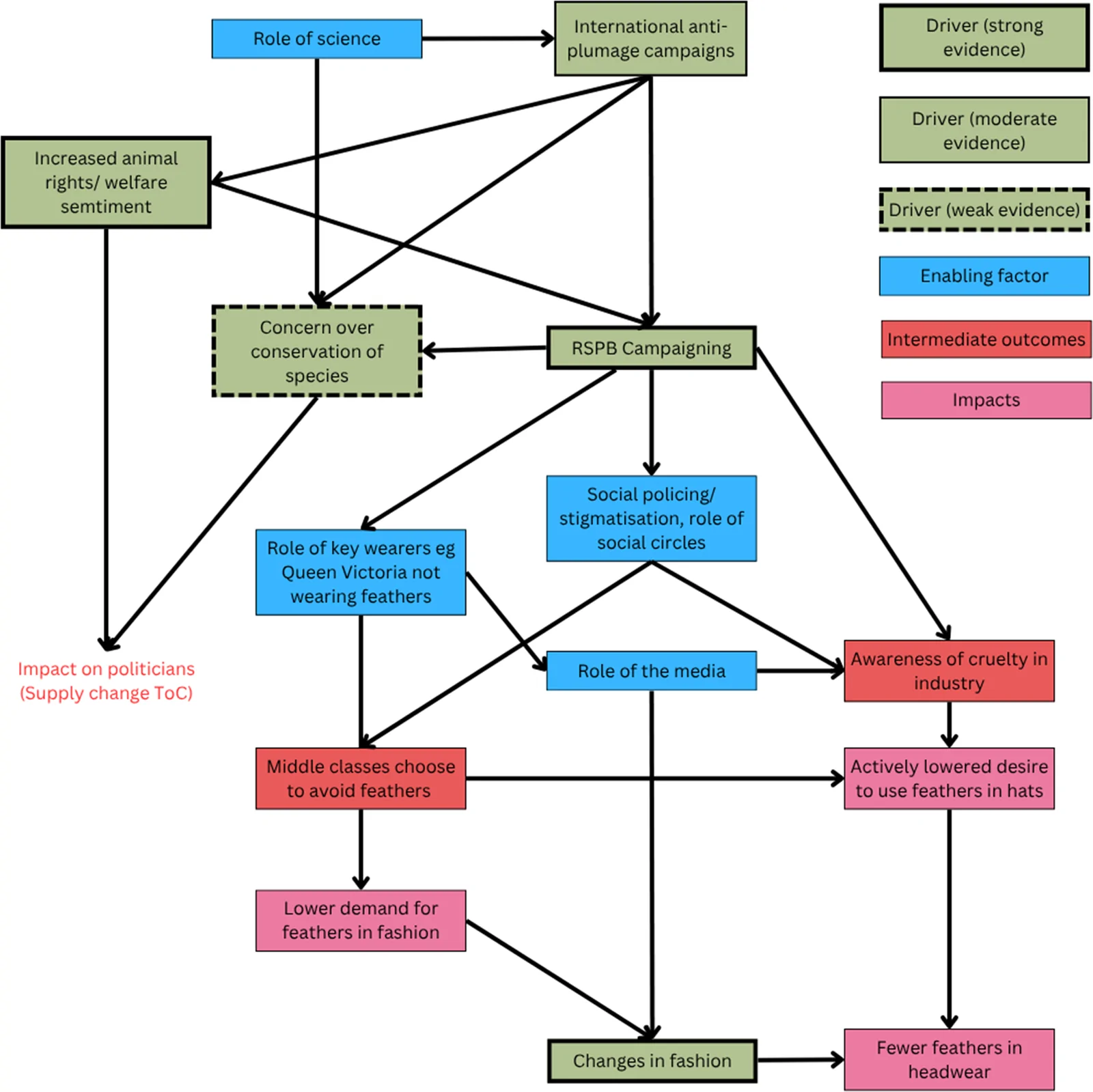
Supply reduction causal pathway. Globalization is included here as a recurring theme discussed by multiple experts as effecting the whole supply system at all points, leaving this unlinked being a reflection of expert ideas.

### 3.2. Contextual causes of change in demand

The period of interest was a time of significant social change. Drivers indirectly affecting the use of feathers in fashion were frequently cited as key causes of changes in feather wearing, including WWI and fashion shifts such as those towards shorter hair and more natural silhouettes, for example, through changing attitudes to corsets.

‘The 1910s is a really voluptuous time in fashion. It's all about S-bend fashion, which is all about corsets … to create this S-bend silhouette. Whereas the 1920s is all about these tubular straight up and down bodies’ (expert I1).

This is somewhat backed by import data and observed trends in feather popularity; however, rebounds in imports subsequent to WWI argue that this event alone was insufficient to kill the trade, as some experts reflected:

‘Post-war there was a great resurgence … The plumage trade had a massive boom straight after the first world war’ (expert O1). ‘I think these changes would have happened anyway, but they're sped up by the wars. Like they're kind of a catalyst … look at what's happening before … women's silhouettes are changing’ (expert M1).

The role or attitudes of women also appeared to be intrinsically linked to change in fashions. Women's employment rates changed significantly during this time as a result of WWI, necessitating more practical fashions [32].

‘Women had more practical lives and [were] working … so they had to be more practical. And so feathers kind of suggest a frippery’ (expert I3).

Fashion cycles were among the highest listed driver, with 10 experts across all stakeholder groups citing this as an important factor. While fashion trends are linked to many other drivers discussed here, the nature of fashion towards change generally, alongside its key role in social living, is highlighted as key cause in the phasing out of feathers as a popular choice [9].

‘The fashion cycle is influenced by all of these things as well, but you know this, the sort of appetite for things going up and down, you know, things coming in and out’ (expert M2).

Contextual factors probably played facilitating roles in the move away from exotic feathers, such as economic changes including the imposition of minimum wage on the feather workforce [33] with lesser evidence for changing attitudes towards the countryside [34].

Figure 5 gives an indication of how these drivers interacted, based on expert account and supporting evidence, to result in a ‘phasing out’ of popular use of exotic feathers in fashion.

**Figure 5.**
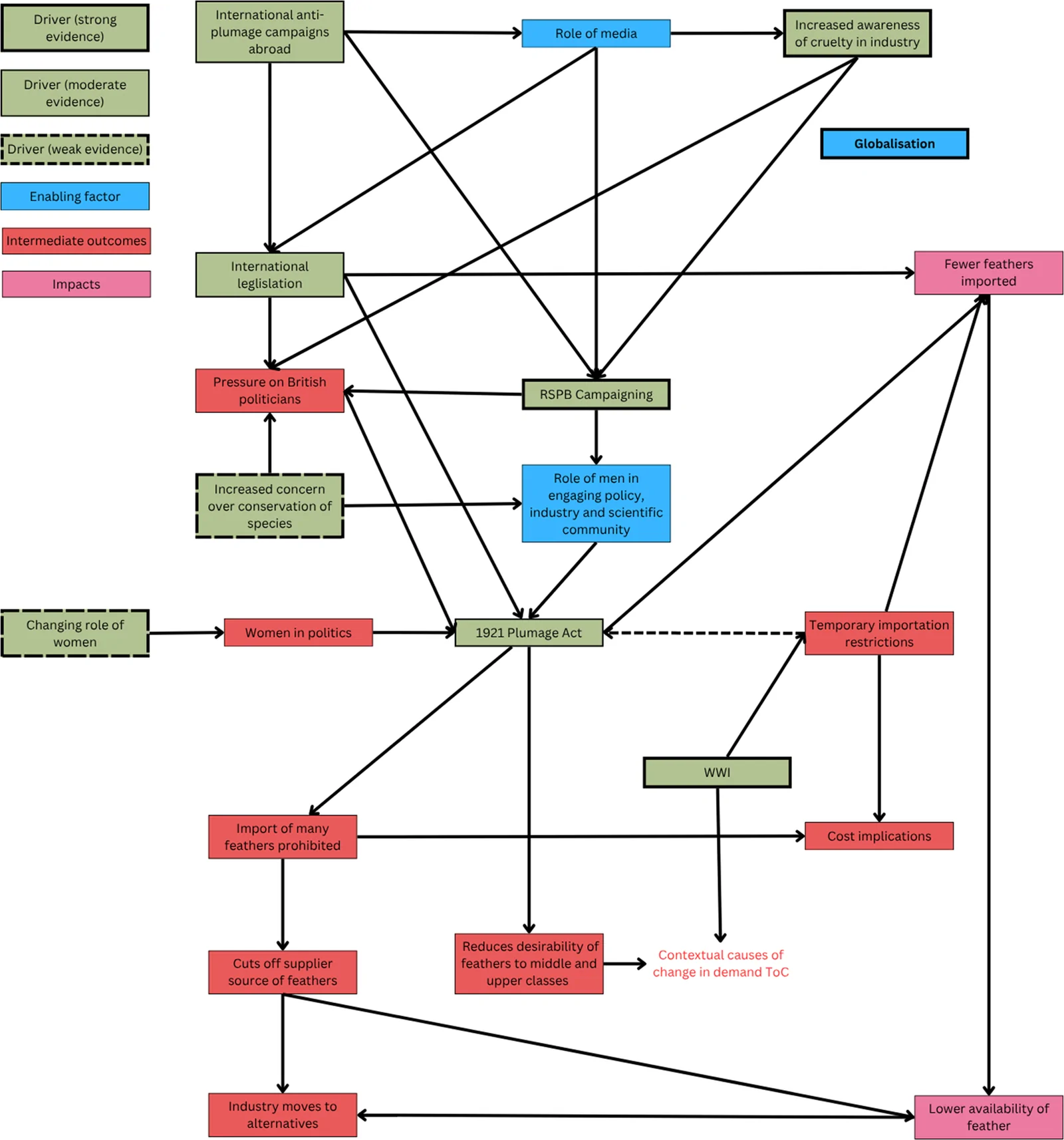
Normative/passive change in demand causal pathway.

### 3.3. Social change in demand

Although broader societal changes have been linked to reduced feather use, this shift was probably catalysed by direct actions at the time aiming to quash this trend. A key player referenced in this change was the RSPB, which actively advocated for women to forgo exotic plumes in their headwear. Methods to stigmatize feather wearing at home and abroad (e.g. through the Audubon Society in the USA) used multiple methods such as shaming women wearing feathers to increase the social cost of wearing the accessory, for example, through letter sending, and awareness spreading of the role of the feather trade in bird cruelty and species decline. The role of ‘key-wearers’ was frequently cited, with influential figures such as Queen Alexandra supporting the anti-plumage cause publicly [29], as well as the role of the media and increasing globalization, acting to proliferate messages of cruelty in the industry and change fashion with increasing efficiency.

Approach of the RSPB in addressing this issue is described as ‘two-pronged’, one looking to impose social pressure on middle- to higher-class women to forgo their plumes, through approaches such as use of prominent social figures and activism; and the other facing the policy issue, aiming to push policy to place barriers to plume import and possession.

Ultimately, five individuals listed the RSPB as having a key role in the decline in plume wearing: three conservation-based experts and two historians. How, exactly, they had an impact received mixed input, with some citing their role in policy change, and interestingly the role of men in this approach:

‘I think this Plumage Act was a defining piece of legislation. I think there was a clear build up to it through the RSPB campaigning. There was a clear build up to it with Queen Victoria and the royal regiments, there was a clear build up to it through shifting changes in society’ (expert C3).

And others, in normative change among women:

‘They were socially powerful women who would invite other women to a tea party and said, ‘isn't this terrible’… I bet most of it was: ‘have you considered joining our cause, my dear?’ And then the woman subtly got rid of her fancy hat’ (expert C5).

When it comes to policy change, the RSPB has stated a lack of enthusiasm in the resulting 1921 Plumage Act; a founder, Etta Lemon, stated ‘it is impossible to say that the Act is a wholly satisfactory one’ (RSPB Annual Report, 2021 in [29]). Ultimately, evidence suggests that this change could not have been brought about by the RSPB without the catalytic effect of drivers such as WWI and the role of fashion itself. However, this group perhaps did best to exploit circumstances to further their agenda when possible, ensuring they had policy suggestions available and public support ready for when opportunity arose for this change to take place to achieve not just policy, but social change.

Interestingly, while women's roles in society was regularly cited as contributing to the decline, the political role of women was less direct. In fact, the RSPB founders were predominantly anti-suffrage [29]. While this may appear to put these two activist societies at odds, links between the campaigns, for example, shared members and the cumulative growing idea of women affecting policy, results in an underlying link between the two. This is perhaps best highlighted by the first female sitting MP, Nancy Astor, who took a role in pushing the Plumage Act through parliament soon after election [29]. The perceived role of women in politics was therefore mixed by experts, ultimately resulting in insufficient support for inclusion in the final ToC.

Active demand reduction ultimately focused on increasing visibility of the cruelty of the trade and, to a lesser extent, the role of the trade in the decline of species. These fed into global campaigns, with media involvement often cited as a key facilitating link to proliferate the cause (figure 6).

**Figure 6.**
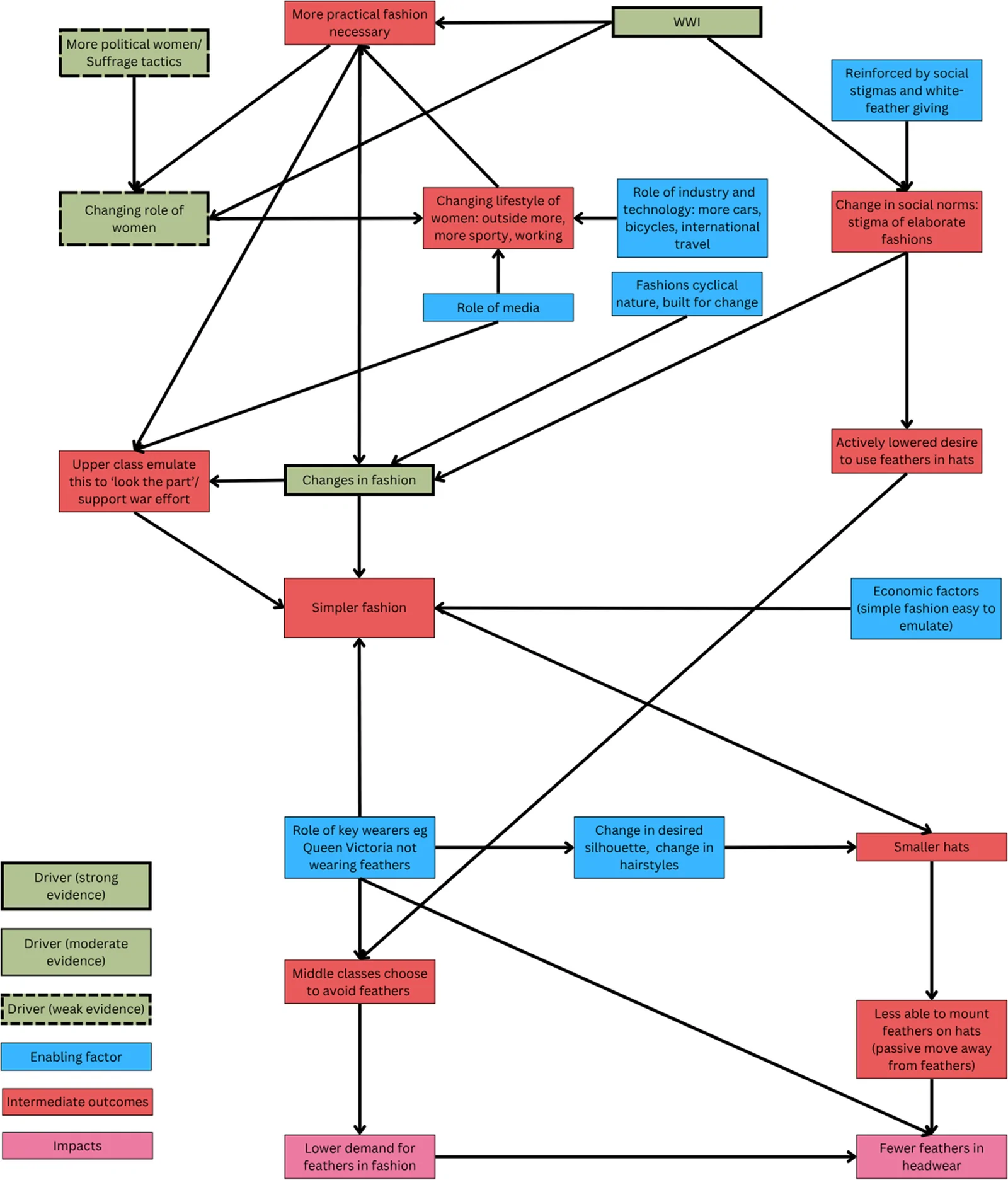
Social active change in demand causal pathway.

Evidence and expert input suggest that RSPB campaigning was indeed key in driving the change away from exotic feather use, showing how advocacy can have behaviour change impact when both consumer and supplier chains are addressed. Given the strong links between drivers and relatively slow progression away from feather use, it is important to note that advocacy is probably effective here in the presence of key facilitating factors including a ‘mood’ of change present throughout this period and significant disruptors to lifestyle such as WWI, alongside the very nature of fashion being changeable.

## 4. Discussion

In this study, we set out to better understand a large-scale behaviour change that resulted in substantial conservation and industry impacts. The change in attitudes towards bird feathers had far-reaching impacts, from observable conservation impact including the rebound in great egret numbers, to the growth in appreciation of wildlife conservation as a national interest, indicated through growing numbers of such organizations and memberships of organizations such as the RSPB (RSPB, n.d.). Through GEM+, we interpret causal relationships supported by expert input, triangulated with evidence and quantitative trends, to suggest that societal behaviour and policy change in the UK be directly linked through lower demand facilitating the eventual passing of the Plumage Act. We also outline the role of global policy reactions affecting demand and supply for this damaging trade (see supplementary material, S4.2), highlighting a positive change for conservation in the face of potentially damaging impacts of globalization.

Evaluating historic behaviour change provides insight into effective project planning for future conservation action. Here, we highlight that, while we can interpret that advocacy played a key role in this positive conservation outcome, interacting drivers came together to ultimately result in a society-level change. This suggests timing of conservation advocacy can be key, and that enabling factors, for example, in this case a significant shifting taste for fashions independently, should be identified as key timings for advocacy. This case also highlights both the importance of interacting with society to achieve behaviour change and with policymakers: while declining use of feathers here predated the Plumage Act, the inception of the Act meant that reigniting the trade was practically inhibited, securing the longevity of the impact. This has implications for policy inception, and demonstrates the importance of continued pressure to implement policy even where need has declined, to prevent recurring damaging trades. This is a very relevant consideration for existing wildlife trades for which countries may consider relaxing their laws where demand wanes.

The decline in feather use has received fair literary interest (e.g. [10,29,35]), but causes of the change remained speculative without systematic evaluation. A useful finding here was the range of support for the concept that RSPB campaigning did indeed show evidence of meaningful impact in conjunction with these societal changes, resulting in strong evidence for plausible causal attribution. Understanding the role of this driver within its contextually complex backdrop was a useful application of GEM+, showing how roles of drivers can be better evaluated and interpreted through this method even in complex settings. This evaluation provides promising support for conservation advocacy, and encourages continual campaigning efforts particularly in times of social change. Given that evaluation plans and evidence-informed ToCs demonstrating project impact are increasingly required for project funding, this case study provides useful supporting evidence for campaigning impact on behaviour change and policy to generate biodiversity positive impacts.

The case of the end of the feather trade provides a unique example of a wildlife trade that has almost completely ceased, and has continued to be largely unpopular through nearly a century. There are global efforts now to end similar trends, such as those for ivory or endangered felid skins, that meet a multitude of challenges from enforcement to cultural acceptance. Learning from a successful example of ending the supply and demand of a trade provides useful information to the range of active and contextual factors needed to effectively end such harmful trades. Efforts to address similarly damaging trades, such as the Asian songbird trade, may find useful insight in this parallel, though culturally different, example.

### 4.1. Impact evaluation methods

In setting out a replicable framework for applying GEM+, we address limitations in existing applications of this method. A key shortcoming identified is the critical use of stakeholder knowledge given that individuals provide most accurate information within their field, and may provide biased responses where they have a stake in the matter [19]. Considering information provided by a milliner on fashion trends or a policy advocate, for example, are unlikely to be equally accurate, and therefore analysis of information by each of these individuals should consider the background of the interviewee. We apply concepts presented in Balanced Evidence Assessment Method [36] to add additional dimensions to evidence analysis, namely, information and source reliability. This allows us to create a method that accounts for individual dimensions of evidence, with the strength of influence of individual accounts more reflective of the accuracy of the evidence they provide.

This method provides a useful tool for historical impact evaluation to better understand actions such as advocacy; however, robustness of conclusions on historical cases is limited by availability of present-day experts. For this reason, conclusions on RSPB impact, where the organization is still active and its impacts observable, may be better understood and documented than the impact of the Plumage Act, where policy experts with lived experience on this Act are no longer alive or professionally active. However, potential biases in knowledge have been somewhat addressed by considering potential stakeholder biases, and by representing expertise across a range of topics. Evaluations will probably be more accurate and representative when carried out soon after a behaviour change is observed; however, historical evaluation provides useful insight into project long-term successes to contribute to future project design.

Uptake of existing impact evaluation methods for conservation more generally has been sparse [37], an issue we hope to address by outlining a replicable impact evaluation method of broad potential use. By outlining and applying GEM+ to an historic example actualized over a century ago, we have illustrated its potential in linking cause and effect in socially complex contexts. This not only highlights the plausible causes of the change but also provides a measure of our certainty around the role of each of these drivers in achieving the change. This provides a basis for other projects to design impact evaluations in socio-ecological contexts where untangling impacts of an intervention can be unclear in the face of multiple interacting factors. We also provide a useful method for approaching contextually difficult scenarios—our example unfolds over several decades and many social changes, making it difficult to approach evaluation in a robust way. GEM+ provides an answer for evaluations in such cases of contextual complexity and data uncertainty, while maintaining a level of robust and transparent output through thorough consideration of reliability of input that is available.

A greater appreciation of a range of impact evaluation methods is key for mainstreaming evaluation in conservation. Global conservation goals such as 30 × 30 have clear necessity for evaluation techniques to meet criteria to prove the ‘effective’ nature of conservation and management planning [38]. To scale conservation to such an extent as is aimed for in global policy, use of a range of methods using a range of evidence types, that can be applied by a range of actors, will be fundamental. Qualitative methods such as these can be taught to a diversity of actors, which is complementary to another key global objective of engaging different sectors in conservation-beneficial behaviours. Furthermore, where conservation efforts intend to expand into new areas and sectors, there will be inevitable baseline data paucity needed for traditional evaluation using counterfactuals. GEM+ can be used in the face of data paucity to infer causality in such contexts.

## Supplementary Material



## Data Availability

Interview transcripts are not available owing to confidentiality requirements. Data used for magazine analysis are available online [39]. Supplementary material is available online [40].
