# Supplementary material for: When feathers fell from fashion: unpacking the end of the UK plumage trade
Source: R Soc Open Sci. 2026 Oct 21;13(10):252133. doi: 10.1098/rsos.252133 (PMC13692776; doi:10.1098/rsos.252133)
Supplement: Supplementary file 1 [file rsos.252133.zip › rsos.252133/rsos252133_si_001.docx]

Supplementary material

**S1: search terms**

Search terms (Google, Google Scholar)

Feather Trade, Bird Feather Trade, Plumage Trade, RSPB Feather Trade

References of driver sources

Boase, T. (2021). *Etta Lemon : the woman who saved the birds*. Aurum Press.

Cowie, H. L. (2021). Murderous Millinery. In *Cambridge University Press eBooks* (pp. 17–54). Cambridge University Press. <https://doi.org/10.1017/9781108861267.002>

Doughty, R. W. (1975). *Feather fashions and bird preservation : a study in nature protection*. University of California Press

House of Commons Debates

House of Lords Debates

Jameson, C. M. (2023). *Finding W. H. Hudson*. Pelagic Publishing Ltd.

Moore-Colyer, R. J. (2000). Feathered Women and Persecuted Birds: The struggle against the Plumage Trade, c. 1860–1922. *Rural History*, *11*(1), 57–73. <https://doi.org/10.1017/s0956793300001904>

Moss, S. (2013). *A Bird in the Bush*. Aurum.

RSPB. (1911). Feathers and facts. A reply to the Feather-trade, and review of facts with reference to the persecution of birds for their plumage. In *Internet Archive (Internet Archive)*. The Royal Society for the Protection of Birds. https://doi.org/10.5962/bhl.title.28010

*RSPB: Our History*. (n.d.). Www.rspb.org.uk. <https://www.rspb.org.uk/about-us/our-history>

Stein, S. A. (2008). *Plumes*. Yale University Press.

Swadling, P., Wagner, R., & Billai Laba. (2019). *Plumes from paradise : trade cycles in outer Southeast Asia and their impact on New Guinea and nearby islands until 1920*. Sydney University Press.

Swan, J., Debo, K., Dirix, E., Marschner, J., O'Neill, A., Van Godstenhoven, K. (2014). *Birds of Paradise: Plumes and Feathers in Fashion: Plumes & Feathers in Fashion - Pluimen & Veren in de Mode*. Antwerp: Lannoo Publishers.

**S2: interviewee characteristics**

S2: Characteristics of experts interviewed, including self-defined areas of expertise (in which experts scored themselves 7 or higher)

| Participant | Stakeholder Group | Industry | High Information reliability |
| --- | --- | --- | --- |
| I1 | Industry | Milliner, Academia, Consultant | Fashion History, Millinery Fashion Trends, British Cultural History |
| I2 | Industry | Feather Worker | Use of feathers in fashion |
| I3 | Industry | Milliner, Costume theatre | Fashion History |
| I4 | Industry | Milliner | Millinery fashion trends, use of feathers in fashion |
| I5 | Industry | Milliner | Fashion history, Millinery fashion trends, Use of feathers in fashion |
| C1 | Conservation | Nature protection, author | Nature protection organisations |
| C2 | Conservation | Conservation organisation | Nature protection organisations |
| C3 | Conservation | Academic, Environmental historian, media | Nature protection organisations, British cultural history, British policy history |
| C4 | Conservation | Public sector, author, nature protection | Nature protection organisations |
| C5 | Conservation | Media, academia, author | Nature protection organisations, British cultural history, British policy history, Use of feathers in fashion |
| M1 | Art Historian | Curator, Arts | British cultural history |
| M2 | Art Historian | Curator, Arts, Fashion | Fashion history, Millinery fashion trends, Use of feathers in fashion, British cultural history |
| M3 | Art Historian | Curator, Fashion history, Museums | Fashion History, Millinery fashion trend, British cultural history, British policy history |
| M4 | Art historian | Fashion historian, academia | Fashion history, Millinery fashion trends, Use of feathers in fashion, British cultural history |
| M5 | Art historian | Hat maker, Curator | Fashion history, Millinery fashion trends, Use of feathers in fashion, British cultural history, British policy history |
| O1 | Other | Journalism | Use of feathers in fashion, Nature protection organisations |

**S3: interview guide**

1. Introduction

Thank you for agreeing to take part in this study.

The goal of my research is to gain a deeper understanding into the changes in use of feathers in fashion in the UK between the late 19^th^ and early 20^th^ century, and, in particular, what drove these changes.

Throughout the interview, I will ask you questions about your background, perception of this change, and what you believe may have been important causes of this change. This will be through open questions and interactive sorting tasks. I will take some notes as we talk.

If any content or question is unclear throughout the process, please let me know and I will make the process as clear as possible.

Please note that if you would like to revise your answers to any question at any time, before or after the interview, you are welcome and encouraged to do so.

2. Part 1

To start with, I would like to know more about your background. Can you please tell me a bit about your current position and any previous positions, experiences, or education you have held that you believe may be relevant here?

*Prompt: How do you think these roles may have provided insight into the topic of feather use in fashion?*

3. Part 2

**2.1** As previously mentioned, I am interested in finding out why fashion changes occurred over this period. I will now show you a figure that roughly demonstrates millinery fashion preferences between 1910 and 1930.

*(Present image)*

You may notice some changes in these images over the time period. How would you describe these changes?

*Prompt: if feathers not focussed on in previous answer: In particular, I am interested in the popularity of feathers in general in this fashion over this entire period. Can you please describe changes you can observe in the ~~use~~ popularity of using feathers in millinery fashion in these images over this period? Do you think use of feathers increased, decreased, or stayed the same in this time?*

In your informed opinion, which drivers do you believe caused this observed change, resulting in the frequency of feather use observed by 1930?

A driver can be described as an event or contextual factor that contributed towards or caused a change.

**2.2** Thank you for your insights. I am interested to learn more about the importance of these drivers of change you have identified. I have your described drivers here on cards. I am now going to present you with a scale which goes from 1 to 7.

I would like you to place each driver along the scale to indicate how important it was in causing the change in feather use in fashion.

7 would indicate that the observed change would not have occurred without this driver; the presence of the driver was fundamental in the change occurring, 1 would indicate the change would have occurred with or without the driver.

The midpoint shows you neither disagree nor agree with the idea that the driver was important for the change.

If you’re not sure how important it was to the change – you’ve just mentioned it as you know it played a role, but you’re not sure to what extent, we can leave that to the side.

*Prompt: For example, if I was asking you about the move to working from home in the UK as an effect, and as a cause or ‘driver’ I had COVID-19, I would, personally, put this as a ‘7’ on the scale.*

Thank you, I’m now going to take a picture/ screenshot of your answers for future reference.

4. Part 3

I also have some additional drivers that I have come across whilst reading about these topics. **present cards**

Which, if any of these, are you unfamiliar with, so unable to comment on the effect of?

Firstly, I am interested to know which, if any, of these you think are not important in this change in fashion?

**remove ‘unimportant’ cards**

As you have not mentioned these cards, that leads me to believe that you think each of these drivers played some role in the observed change. As you did previously with your suggested drivers, can you please place these on the 1-7 scale of importance towards this change?

*Draw this out as they do it.*

I have drawn out a rough map for what I believe you are saying. Does this look like what you were describing? Is there anything you want to add or change?

5. Do I have any follow-up questions on fashion changes they mentioned? Eg, they may have mentioned particular fashions such as ostrich vs other.
6. Part 4

Finally, I want to circle back to your expertise. Could you please indicate below your self-perceived level of expertise on each of the following topics? Please note, these topics are referring to my time period of interest.

| **Topic** | **1** | **2** | **3** | **4** | **5** | **6** | **7** | **8** | **9** | **10** |
| --- | --- | --- | --- | --- | --- | --- | --- | --- | --- | --- |
| **Fashion History** |  |  |  |  |  |  |  |  |  |  |
| **Millinery Fashion Trends** |  |  |  |  |  |  |  |  |  |  |
| **Use of feathers in fashion** |  |  |  |  |  |  |  |  |  |  |
| **British Cultural History** |  |  |  |  |  |  |  |  |  |  |
| **British Political/ Policy History** |  |  |  |  |  |  |  |  |  |  |

7. Conclusion

**Thank you** for your time, your contribution is incredibly helpful to me. I will now take your responses and analyse them, and hope to gain an overall understanding of what drove these changes and why. **If you have any follow up questions, please don’t hesitate to email me**. Additionally, **if there’s anything you would like to add** at a later date, please feel free.

**I will send you my initial results as to what your opinions ‘tell’ me once I have analysed my findings, which you will be welcome and encouraged to comment on.**

Before I end the interview, is there **anything you would like to add on the topic of bird feathers in fashion**?

**Do you perhaps know of/ could suggest anyone else who may be interesting/ useful to interview on this topic? Or any useful resources?**

**S4.1: additional driver evidence**

| **Driver** | **Evidence** |
| --- | --- |
| **Fashion Trends**  That the decline was observed due to change in women’s fashion preferences | **Figure 1, 2, 3, 5**  As your Lordships are doubtless aware, there has been a considerable change in fashion in ladies' adornment within the last few years, not confined to hats but extending to other parts of the female dress. Whether by reason of the Act of 1921, or from some other occult reason, there is no doubt that feather hats are not so popular as they were. There are numbers of hats, I am assured, both of the cheaper and of the more expensive kind, which are now worn, in which feathers play very little part. Feathers have been superseded by other forms of adornment, such as flowers and other things, but the fact remains that there are still a large number of feathers of this prohibited kind openly sold in some of the principal shops… change in fashion has lessened the demand for these most valuable plumes traders have still some plumes that they bought before 1921 and which they are willing to sell… There is nothing so hard and so cruel as the vanity and the cruelty of fashion, and that is one of the things that we desire to stop… the fashions have changed and there is really no demand at the present moment for plumage of that kind. The plumage trade is at present almost dead…  believe it to be true that the fashion of women wearing plumage in their hats or in any other way is becoming very unusual (HoL 22/03/1928).  I hold in my hand an important exhibit—in contradistinction to my noble friend's exhibit the other day—of spring advertisements and other advertisements displayed by no fewer than twelve of our largest firms that deal with these matters, in which they offer these feathers for sale, either as plumes or in hats…  I hope I have shown that such a trade exists, that it is going very strong, that there is a considerable demand for these feathers (HoL 8/5/28)   it was quite clear that, as the result of change of fashion or as the result of the prohibition or as the result of the emotion which had arisen in females' minds of possible cruelty by wearing these feathers, the business fell almost dead and nobody wore feathers… women's fashions had entirely changed and there was no necessity to bring the feathers in. (HoL 26/4/28)  I have said that there has been a diminution of the wearing of these feathers, but from time to time there is a curious recrudescence of a demand for them. Four or five years ago a considerable number of the reputable shops that deal in these things had in their catalogues a considerable number of advertisements offering egret feathers for sale, mainly in hats…As naturally follows when fashion changes, the price of these feathers has steadily fallen. If it were necessary, I could give figures to show that there has been a tremendous fall in the price of this class of feathers during the last few years. (HoL 8/5/28)  [on women continuing to wear long skirts despite risk of tuberculosis] “but it has become the custom, and the votaries of the fashion will continue to obey the absurd and loathsome dictates as long as the fashion exists…it is difficult to account for the prevalence of the trailing skirt or any other fashionable folly…nine-tenths of the women who wear these trailing dresses do so from no other motive than that it is the fashion, which they are bound to follow”. (The Queen 3/11/1900)  Plumage is to be very specially dedicated to these same little hats, and fortunately one may safely say that in the majority of instances they will not be composed of rare burs species specially slaughtered for this end. Nowadays, the plumage of geese, chickens, pigeons, seagulls and many wild birds killed for eating purposes van be so dressed and dyed that they represent every known [] fowl. (The Queen, Millinery of the Moment, 28/10/1916)  The majority of women seem to find small-brimmed hats more practical for everyday use…the modern milliner having a distinct flair for producing artistic effects…mostly brought about by the judicious use of lace (The Queen, 21/10/1923)  Glycerined ostrich feather continue to be used for both big and small hats (The Queen, 15/11/1923)  Easier to wear are the afternoon hats with tiny front brims and charming nose-length veils; and these hats have the great attraction of making their wearer look quite different from the open-air, golf-and-tennis playing creature of the morning (The Queen, 7/1/1928)  Between 1886 and 1896, shifts in fashion caused the value of ostrich feathers to plunge by 75% (Stein, 2008)  Fashion preferences, 1931 article: “The claim of women to equality with men is understandable, but that so many of them should wish to imitate the appearance of the less beauteous sex is not so easy to understand. Yet the whole tendency of feminine fashion in recent years has been in this direction. It began with bobbing, and reached its logical hirsute conclusion in the Eton crop.” ([Daily Sketch, 1931](https://www.tandfonline.com/doi/full/10.1080/09523367.2012.675206?casa_token=7PqjKRCXh4oAAAAA%3AWSHQ-U9CSjeG1KgBJ2L6eDyZviyiZT5jP8t8YBNjkfoaCgDxFijF3cAd1gm0jr3vEJk6Cil6SLkl_g#d1e467))  1906: Queen Alexandra stated she would not use osprey.  1915: Famous actress Irene Castel cut her hair short, into a bob. |
| **Export legislation abroad effecting supply**  That these legislations caused a decline in exotic feather use in the UK | **Figure 4; International legislations timeline**  The twenty-one Colonies and Possessions to which I have referred all have laws preventing the exportation of the plumage of certain birds, and the Committee at present sitting is considering the possibility of preventing the importation into the United Kingdom of the plumage of those special birds. (HoC, 22/2/1911)  There was also at that time [of passing 1921 Plumage Act] a strong desire in this country to support legislation that was in progress in several of the Dominions, as for instance in Canada, in Australia, in India and it parts of Africa, with the object of prohibiting the export of plumage from those countries, in the interests of bird protection… there is in a minor way the Imperial side of the matter. Australia has very strong views about this.. Tasmania, India, and parts of Africa have all prohibited exports, and this Bill would enable their efforts to meet with further success. (HoL 22/03/1928) |
| **Anti-plume wearing campaigns abroad**  That these campaigns had an impact on feather-wearing in the UK | Audubon Societies (in the US) a regular feature in Bird Notes and News (RSPB publication), as well as RSPB Annual Reports  In 1881 protests had commenced in India over the killing of green parrots and other insect-eating birds for their plumes, leading to Wild Birds Protection Act of 1887… When the Society for the Preservation of the Wild Fauna of the Empire founded (1903), similar laws to those enforced in India were also implemented in many other British colonies (doughty, 1975). This group, for example, was involved in the 1908 Papua legislation (see timeline, Swadling 1908).  Images for the RSPB 1911 Sandwich board campaign taken from Australian publication Emu, taken by A. H. E Mattingley who was made an Honorary Life Member of the RSPB (Boase, 2021) |
| **More women in politics/ suffragette impact**  That the increase in women’s role in politics and the actions of the suffragettes has an impact on women’s feather wearing | Nancy Astor was the first MP in parliament, 1919. She became involved in pushing through the Plumage Act.  [many] members/ founders if the SPB were anti-suffrage.  RSPB was a female-founded movement, with men only allowed as ‘honorary members’ for its formative years (Clarke, 2004). Suffrage campaigners often seen with feathered hats.  The annual meeting of the Royal Society for the Protection of Birds…Queen Alexandra gave full permission…to use Her Majesty’s name in any way the Duchess though best to conduce the protection of birds…If ladies who endeavour to take men’s places in the world of politics would give up the wearing of articles obtained by unnecessary cruelty, they would do more to extent their influence than by any possible agitation for women’s rights. IF woman shows herself unjust, thoughtless, and cruel in dealing with the things which come absolutely under her own dominion, how is it to be supposed she would prove herself invariably beneficent when dealing with other people and their affairs! (The Queen, 31/3/1906)  Emmeline Pankhurst: “our members are absolutely single minded” (Boase, 2021, pg 192)  Miss Clara Mordan wrote to Votes for Women, 1909: I feel myself impelled to put a plea for the birds, and to suggest that women should take the opportunity of dissociating themselves from Murderous Millinery” Clara was an important donor to the [WSPU] cause.  WPU member: “Perhaps it is because we are fighting so hard for liberty, that so few of our members transgress in this way against the birds” (Boase, 2021, pg 190)  Clara Donated £100 to the WSPB a month before an exhibition in which ‘no murderous millinery…is in the official photograph’ (Boase, 2021, pg 194) |
| **WWI**  That WWI caused a decline in exotic feather wearing | **Figures 1, 2, 3, 4, 5, international legislation timeline**    1917 wartime importation prohibitions: included plumage. (HoC, 22/2/1911).  More women in the workforce: Women’s employment rates increased during WWI, from 23.6% of the working age population in 1914 to between 37.7% and 46.7% in 1918 ([Braybon 1989, p.49](https://www.striking-women.org/module/women-and-work/world-war-i-1914-1918)). |
| **Economic changes**  That the change was caused by economic changes in the UK |   Source: [Wardley-Kershaw & Schednk-Hoppe, 2022](https://www.mdpi.com/2409-9252/2/4/28#:~:text=The%20global%20economic%20fallout%20from,in%20Britain%20during%20these%20periods.)  Great Labour Unrest of 1911-1914 (large numbers of strikes)  Creation and enforcement of a minimum wage scale for London’s blue-collar feather workforce post 1914 (Stein, 2008)  The advertisements are taken from *Tatler* magazine for the years 1925, 1930 and 1935. The increasing proliferation of advertisements in *Tatler* during the sample years directly reflects the general growth in expenditure on leisure during these years. Between 1925 and 1935 the number of pages in each edition of *Tatler* dedicated to advertising generally increased by 10%, number of ads with sportswomen increased over this time ([Skillen 2012](https://www.tandfonline.com/doi/full/10.1080/09523367.2012.675206?casa_token=7PqjKRCXh4oAAAAA%3AWSHQ-U9CSjeG1KgBJ2L6eDyZviyiZT5jP8t8YBNjkfoaCgDxFijF3cAd1gm0jr3vEJk6Cil6SLkl_g#d1e223)).  Languishing in the hold of several ships in the Victoria and Albert docks that sweltering August [1911]…were 618 tons of bird-skins and feathers/ the striking dockers had brought London, and its commodity auctions, to a standstill. (The Spectator, 1911 in Boase, 2021) |
| **Increased access to/ time spent in the countryside eg more bicycles**  That women being more active in the countryside, for example through increased use of bicycles, caused a change | The term bird-watching first coined in 1901 – using field instead of museum studies (Selous, 1901).  First national bird census in Britian: 1928 (Guida, 2019) |
| **Plumage Act**  The Plumage Importation (Prohibition) Act passed in 1921 prohibited the import (but not sale) of (most) exotic plumes into the UK.  That this caused the decline in feather use in the UK | **Figure 3, 4, Timelines**    (Doughty, 1975)  [Prohibited Plumage(Seizures) - Hansard - UK Parliament](https://hansard.parliament.uk/Commons/1926-03-30/debates/01cad420-2740-4a4b-bd95-22795b483de3/ProhibitedPlumage(Seizures)) (30/03/1926)  What I should like to tell your Lordships is that since the passing of the Act there has undoubtedly been a great deal of smuggling of this forbidden plumage into the country…That forbidden plumage is being openly sold in some of the leading shops in this country and is purchased by women who, I suppose, have theories of their own as to what they should wear… The Customs report that:— "There is no reason to suppose that plumage is being smuggled to any extent.” (HoL 22/03/1928)  it was quite clear that, as the result of change of fashion or as the result of the prohibition or as the result of the emotion which had arisen in females' minds of possible cruelty by wearing these feathers, the business fell almost dead and nobody wore feathers…Dealing with trade importations—and that is really the subject upon which we are engaged this evening—your Lordships will have observed that the numbers have fallen from 48 in 1925 to 17 last year. (total number of what are technically called seizures by the Customs during the years 1925, 1926 and 1927) (HoL 26/4/28)  I should like to say here—and I think I can speak on behalf of the members of my Committee as well—that the Act of 1921 has been a distinct success in many respects. It has undoubtedly very much diminished the import of birds' plumage, it has discouraged the wearing of plumes and it has appealed also to humane feeling in regard to these birds. (08/05/1928)  When Britian passed the Plumage Act, the plume trade went into decline. By the mid 1920s the price paid for plumes had fallen (Cheesman, 1938 in Swadling, 2019). |
| **Increase in concern over conservation of species**  That there was an increased appreciation of need to protect species, causing a decline in feather-wearing | 49,000 ounces of osprey plumes were placed on the London market, representing 297,000 white herons, I think it is high time this House interfered with the importation of these plumes. Otherwise we shall certainly, in a very short time, exterminate these beautiful birds. (HoC, 22/2/1911)   the Act of 1921 was passed in response to a very widespread public feeling in this country and in many of our Dominions and Possessions, which strongly condemned and deplored the gradual extermination of a great number of beautiful birds…The truth is that there has been a very large amount of smuggling, some of it successful and some unsuccessful…  (HoL 22/03/1928)  it was quite clear that, as the result of change of fashion or as the result of the prohibition or as the result of the emotion which had arisen in females' minds of possible cruelty by wearing these feathers, the business fell almost dead and nobody wore feathers. (HoL 26/4/28)  Almost the first thing that the Committee did was to decide to exclude at all events the bird of paradise and the egret from the Schedule on the ground that the demand for these birds, which were among the most ornamental, was diminishing their numbers, and that in the case of the egret the acquisition of the feathers was also bound up with considerable cruelty. (HoL 08/05/1928) |
| **RSPB Campaigning**  The Royal Society for the Protection of Birds campaigned for women to stop wearing feathers in their hats, and also campaigned for the implementation of legislation to regulate the trade. | **Figure 5, RSPB Timeline**  *Methods to appeal to women to not wear feather included: lectures; newspaper articles; sandwich board campaign of 1911 (showing destruction of herons and starving chicks); letters to individuals seen wearing feathers to discontinue use. Methods could include zoological information, appeal to sense of morality, or be actively shameful*.  RSPB included in Plumage Act advisory committee.   I can assure your Lordships that the society of which I am President, the **Royal Society for the Protection of Animals**, and every humane society in England, Ireland, Scotland and Wales, has been anxiously awaiting the passing of this measure (HoL, 21/06/21)  I should like to ask the Government if they are prepared to undertake that one representative of the Royal Society for the Protection of Birds and one representative of the Royal Society for the Protection of Animals should sit upon that Committee…When the great efforts which have been devoted to getting this Bill through Parliament have exhausted themselves (HoL 23/06/1921)  I have also had resolutions sent to me from branches of the Royal Society for the Prevention of Cruelty to Animals all over the country, strongly supporting this Bill (HoL 22/03/1928)  The [RSPB] has sent me two of their leaflets…I have just enrolled three new members of the Society (The Queen, 14/7/1894)  Queen Alexandra gave “full permission to use her name in any way” – evidence society was not a ‘faddist minority’ (Doughty 1975)  SPB branch contacted local milliners ‘asking their cooperation in the preservation of  our fast-decreasing foreign birds by refraining from exhibiting egrets, herons and paradise plumes in their models, and by not suggesting their use when not expressly ordered’. Three of the milliners of Southport supported the SPB’s appeal… personally they would gladly cease the supply (Liverpool Mercury in Cowie, 2021) |
| **Increased animal rights/ welfare sentiment** | those of you who are familiar with the cruelties that have been perpetrated in connection with the plumage trade will wish this Bill well…evidence about them is that the traders cut, the wings off many of the living birds and left them to die in agony while suffering from these wounds…I am certain that no one whom I am addressing would permit, for a single moment, the infliction of needless cruelty and suffering upon any form of the living creation which has not some full and effective compensation (HoL, 21/06/21)  The original Plumage Act was to seeking to protect animals or birds from the unthinking cruelty of man (HoL 22/03/1928)  it was quite clear that, as the result of change of fashion or as the result of the prohibition or as the result of the emotion which had arisen in females' minds of possible cruelty by wearing these feathers, the business fell almost dead and nobody wore feathers. (HoL 26/4/28)  Almost the first thing that the Committee did was to decide to exclude at all events the bird of paradise and the egret from the Schedule on the ground that the demand for these birds, which were among the most ornamental, was diminishing their numbers, and that in the case of the egret the acquisition of the feathers was also bound up with considerable cruelty. (HoL 08/05/1928)  Milliners shifted to ‘artificial osprey’ and claimed that feathers were collected after they had been shed by the bird in response to cruelty concerns over practice of feather collection methods (eg Cowie, 2021)  Letter from Queen Alexandra to RSPB: “Her Majesty never wears osprey feathers herself, and will certainly do all in her power to discourage the cruelty practised on these beautiful little birds” (Boase, 2021) |
| **Changing role of women – cultural shifts** | More women in the workforce: Women’s employment rates increased during WWI, from 23.6% of the working age population in 1914 to between 37.7% and 46.7% in 1918 ([Braybon 1989, p.49](https://www.striking-women.org/module/women-and-work/world-war-i-1914-1918)).  Increase in women’s sporting advertisements including simpler fashions;  “The advertisements are taken from *Tatler* magazine for the years 1925, 1930 and 1935. The increasing proliferation of advertisements in *Tatler* during the sample years directly reflects the general growth in expenditure on leisure during these years. Between 1925 and 1935 the number of pages in each edition of *Tatler* dedicated to advertising generally increased by 10%”, number of ads with sportswomen increased over this time (eg short hair; [Skillen 2012](https://www.tandfonline.com/doi/full/10.1080/09523367.2012.675206?casa_token=7PqjKRCXh4oAAAAA%3AWSHQ-U9CSjeG1KgBJ2L6eDyZviyiZT5jP8t8YBNjkfoaCgDxFijF3cAd1gm0jr3vEJk6Cil6SLkl_g#d1e223))  Easier to wear are the afternoon hats with tiny front brims and charming nose-length veils; and these hats have the great attraction of making their wearer look quite different from the open-air, golf-and-tennis playing creature of the morning (The Queen, 7/1/1928) |
| **Bird extinctions reducing supply** | There are twenty-one of our Colonies and possessions in favour of taking some immediate steps to prevent the sale or importation to this country of the skins and plumage of certain rare and beautiful birds. These birds are likely to become extinct in a very few years, in fact we may say that for the last ten years one or two species of rare birds have become extinct every year, and looking at the urgency of this question…All we ask is that in addition we shall consider the question of preventing the importation into England of certain other birds which are likely to become extinct during the next few years. (HoC, 22/2/1911)  Thirteen out of the 18 species of humming birds have been exterminated in Trinidad alone (HoL, 21/06/21)  Trade responsible for 95% decline in North American Great Egrets [(All About Birds, 2024](https://www.allaboutbirds.org/guide/Great_Egret/lifehistory)). Severe losses of Snowy Egret from the trade ([All About Birds, 2024b](https://www.allaboutbirds.org/guide/Snowy_Egret/lifehistory))  Walter Goodfellow _ having travelled in the area for several years _ believed that the extermination of birds of paradise was imminent because many species had specialised ranges, and shooters had begun to penetrate deeper into the interior for four stock birds (Charles F Downham, The Feather Trade *in* Doughty, 19750)  Some 12–15 million small herons and 3 to 4.5 million large ones were killed for the plume trade in Argentina, Brazil and Venezuela (Swadling, 2019). |

**S4.2: driver evidence supporting figures and quantitative trends**

*S4.2.1: Indication of millinery fashion trends adapted* *from Amphlett, H. (2003). Hats: a history of fashion in headwear. Dover Publications, enhanced with StarryAI*

*.*

*S4.2.2: Indication of millinery fashion trends based on* Amphlett, H. (2003). Hats: a history of fashion in headwear. Dover Publications, *enhanced with*

*To outline trends of feather use in women’s fashion in the time period of interest, fashion magazines from this time period were investigated as a primary data source. The Queen, as a magazine running throughout the entire period, with weekly issues available, was investigated to provide an indication of preferences for feather wearing in higher class society. This was used as an indicator of overall popularity of feather wearing at the time. Each fourth magazine between 1891 and 1930 was analysed for feather references including imagery and wording, with page coverage of each instance approximated and categorised as either an advertisement for the feathered item or non-advertisement. Strong inter-collector agreement in categorisation and size approximation was achieved prior to beginning data collection (K=0.80+- 0.07). Figure S4.2.3 demonstrates feather content coverage trends found in this magazine.*

*S4.2.3: Proportion of The Queen magazine issue coverage with 1) advertisement and 2) non-advertisement feather imagery and text (1891-1930). Points show mean ad/ non-ad cover for that year, ribbons show interquartile ranges of cover ranges*

*S4.2. 4: Importation (in lbs) of feathers, divided into ostrich and ornamental (included exotic plumage) into the UK, 1900-1930*

|  | Society for the Protection of Birds | Fancy feather |
| --- | --- | --- |
| 1889 | 20 | 223 |
| 1890 | 6 | 306 |
| 1891 | 212 | 179 |
| 1892 | 239 | 132 |
| 1893 | 204 | 242 |
| 1894 | 136 | 176 |
| 1895 | 281 | 182 |
| 1896 | 205 | 135 |
| 1897 | 255 | 99 |
| 1898 | 186 | 210 |
| 1899 | 224 | 177 |
| 1900 | 183 | 143 |
| 1901 | 198 | 152 |
| 1902 | 228 | 130 |
| 1903 | 281 | 102 |
| 1904 | 233 | 100 |
| 1905 | 301 | 48 |
| 1906 | 457 | 86 |
| 1907 | 274 | 81 |
| 1908 | 378 | 137 |
| 1909 | 318 | 142 |
| 1910 | 254 | 165 |
| 1911 | 267 | 100 |
| 1912 | 245 | 104 |
| 1913 | 284 | 233 |
| 1914 | 225 | 168 |
| 1915 | 209 | 72 |
| 1916 | 134 | 52 |
| 1917 | 136 | 23 |
| 1918 | 60 | 14 |
| 1919 | 110 | 30 |
| 1920 | 72 | 50 |
| 1921 | 113 | 60 |
| 1922 | 134 | 32 |
| 1923 | 147 | 30 |
| 1924 | 216 | 35 |
| 1925 | 244 | 26 |
| 1926 | 323 | 14 |
| 1927 | 309 | 4 |
| 1928 | 216 | 30 |
| 1929 | 211 | 7 |
| 1930 | 228 | 8 |

S4.2.5: Occurrences of the phrases "Society for the Protection of Birds" and "Fancy Feather" In British Newspapers, 1889-1930 (Source: British Newspaper Archives)

**International plumage import/ export legislation timeline:**

<https://time.graphics/line/933772>

**RSPB: Key events timeline**

<https://time.graphics/line/933737>

**S5: driver supporting evidence grading sheet**

| **Driver** | **Score**  **1: Evidence strongly disagrees that driver played role**  **2: Evidence disagrees**  **3: Evidence neither supports nor refutes**  **4: Evidence supports**  **5: evidence strongly supports** |
| --- | --- |
| **Fashion Trends** |  |
| **Export legislation abroad effecting supply** |  |
| **Anti-plume wearing campaigns abroad** |  |
| **More women in politics/ suffragette impact** |  |
| **WWI** |  |
| **Economic changes** |  |
| **Increased access to the countryside eg more bicycles** |  |
| **Plumage Act** |  |
| **Increase in concern over conservation of species** |  |
| **RSPB Campaigning** |  |
| **Increased animal rights/ welfare sentiment** |  |
| **Changing role of women – cultural shifts** |  |
| **Bird extinctions reducing supply** |  |

**S6: additional information on driver elimination rationale**

*Driver eliminated as a result of elicitation process*:

Where no individuals place a driver at the threshold level along the scale to deem it important to the change, given the diverse representation of expertise that should be present among the panel, this provides confidence that it is extremely unlikely that the driver played a key role in the impact of interest. The driver is removed from subsequent evaluation.

*Low levels of confidence in driver impact*:

Where a stakeholder group with low source reliability list a driver as important, and this is not backed by subject experts with high information reliability, we cannot be confident in this information provided. Whilst there is value in investigating why this belief is held by an entire stakeholder group, significant outside information would be required to substantiate and validate these claims.

Where a driver is placed at a high rating by a single individual that has both high source and information reliability, there is a risk of error being introduced by the input of a single individual. For this reason, groups are considered substantially more reliable than individuals (Burgman, 2016). Without substantiation from information external to interviews, individual testament would be insufficient in these cases to justify the conclusion that drivers with these levels of support played a key role.

*Moderate levels of support for driver role:*

Where one group agrees that the driver was important in the change, however this group is expected to have some underlying bias on the subject of the driver, and this is supported by individuals within or outside of this group also ranking the driver highly with high information reliability, confidence in the driver is improved. Given that motivated reasoning may influence high ratings here in groups with pre-existing affiliations with the driver, this may cause inflated perceived importance of the driver, even in experts (Perez, 2014). Therefore, even though group consensus is reached and information provided is from an informed source, further support from unbiased sources to account for this bias is desirable to increase confidence that conclusions drawn are not an artifact of information informed by biased opinions.

Where multiple individuals with differing expected attitudes towards the changes (ie supportive and unsupportive) rank the driver as important, but single group agreement is not achieved, this also provides a higher level of confidence in the driver than single-expert statement where high information reliability is present for at least one individual. However, this scenario begs the question of what caused single group agreement not to be achieved, probing further investigation for stronger confidence.

*High levels of support for driver role*:

Here, all three factors are satisfied: driver is ranked as important by at least two groups, at least one subject expert with high source reliability, and at least one individual with high information reliability. Where two groups of opposing views on the impact (ie supportive or unsupportive of the change) rank the driver highly, this provides additional confidence in individual judgement. Through this, information acquired is expected to be accurate and reliable based on definitions of evidence weighting and information reliability (Christie et al., 2023). Given that a diverse range of individuals are consulted as a part of this process and expertise should be represented across all topics, we would anticipate incredibly low likelihood of a driver being categorised into this level of confidence as an artefact of error or bias.
